# Supplementary material for: Artifacts in photoacoustic imaging: Origins and mitigations
Source: Photoacoustics. 2025 Jul 5;45:100745. doi: 10.1016/j.pacs.2025.100745 (PMC12305209; doi:10.1016/j.pacs.2025.100745)
Supplement: MMC S1 — Assumptions that are made during the PAI process, and list of the sources, causes, and effects of the artifacts. [file mmc1.pdf]

Supplementary information

**Modeling assumptions: light and sound physics**	
**L1**	Fluence and radiant exposure are not spatially varying.
**L2**	Fluence does not depend on absorption.
**L3**	Fluence is wavelength-independent.
**L4**	All the absorbed energy is translated into an acoustic wave.
**S1**	Sound waves do not decay with depth.
**S2**	The sound speed is uniform in tissue.
**S3**	Sound waves are not scattered in tissue.
**S4**	Sound waves behave independently of their frequency.
**Data assumptions: excitation and detection hardware**	
**H1**	There is sufficient data for exact image reconstruction (spatio-temporal sampling, frequency bandwidth).
**H2**	Laser power is constant for each pulse.
**H3**	The input wavelength is known perfectly.
**H4**	Radiant exposure is uniform.
**H5**	Measurements are free of noise.
**H6**	Light pulse-length is sufficiently short.
**H7**	Detectors are perfectly directional.
**H8**	No signals are measured that have an origin out of the imaging field of view.
**Experimental assumptions**	
**E1**	Acoustic coupling between device and the subject is perfect.
**E2**	Subject motion does not compromise image quality.
**E3**	Tissue properties and detector sensitivity are not affected by temperature and remain constant.
**E4**	Subject preparation or medication does not confound the target imaging biomarkers.
**E5**	There exist no patient-specific confounders (such as skin tone, BMI, sex, age).

Table 1: Assumptions that are made during the PAI process

Source	Cause	Violated Assumptions	Artifact Effect					
			Dislocation	Blurring	Clutter	Signal loss	Signal Change	Splitting
Patient	**Patient Movement**	E2	X	X				
	**Patient Preparation**	E4			X		X	
Light-Tissue Interactions	**Fluence Decay**	L1, L2, H4				With depth		
	**Spectral Coloring**	L3, H3, H5, E5, H2					Between wavelengths	
	**Out-of-plane Absorption**	H8			X			
	**Laser Power Variation**	H2, H3, E3					X	
	**Long Pulse Duration**	H6		X				
The PA effect	**PA Efficiency**	L4, E3					X	
Sound-Tissue Interactions	**Sound Speed**	S2, S3, E1	X	X				X
	**Acoustic Reflections**	S3, S4, E1			X			
	**Acoustic Attenuation**	S1, S3, S4				With depth		
Signal Detection	**Limited View**	H1			X	X		
	**Sparse View**	H1			X	X		
	**Detector Directivity**	H7			X	X		
	**Limited Temporal Sampling**	H1		X	X	X		
	**Limited Frequency Response**	S4, H1		X	X	With spatial frequency		
	**Measurement Noise**	H5, E3				At low SNR	X	

Table 2: List of the sources, causes, and effects of artifacts typically encountered in PAI.
